# Fungal communities associated with almond throughout crop development: Implications for aflatoxin biocontrol management in California

**DOI:** 10.1371/journal.pone.0199127

**Published:** 2018-06-20

**Authors:** Alejandro Ortega-Beltran, Juan Moral, Ryan D. Puckett, David P. Morgan, Peter J. Cotty, Themis J. Michailides

**Affiliations:** 1 Department of Plant Pathology, University of California Davis, Davis, California, United States of America; 2 Kearney Agricultural Research and Extension Center, Parlier, California, United States of America; 3 USDA-ARS, School of Plant Sciences, University of Arizona, Tucson, Arizona, United States of America; University of Nebraska-Lincoln, UNITED STATES

## Abstract

Interactions between pathogenic and nonpathogenic fungal species in the tree canopy are complex and can determine if disease will manifest in the plant and in other organisms such as honey bees. Seasonal dynamics of fungi were studied in an almond orchard in California where experimental release of the atoxigenic biopesticide *Aspergillus flavus* AF36 to displace toxigenic *Aspergillus* strains has been conducted for five years. The presence of the vegetative compatibility group (VCG) YV36, to which AF36 belongs, in the blossoms, and the honey bees that attend these blossoms, was assessed. In blossoms, *A*. *flavus* frequencies ranged from 0 to 4.5%, depending on the year of study. Frequencies of honey bees carrying *A*. *flavus* ranged from 6.5 to 10%. Only one *A*. *flavus* isolate recovered from a blossom in 2016 belonged to YV36, while members of the VCG were not detected contaminating honey bees. Exposure of pollinator honey bees to AF36 was detected to be very low. The density of several *Aspergillus* species was found to increase during almond hull split and throughout the final stages of maturation; this also occurred in pistachio orchards during the maturation period. Additionally, we found that AF36 effectively limited almond aflatoxin contamination in laboratory assays. This study provides knowledge and understanding of the seasonal dynamics of *Aspergillus* fungi and will help design aflatoxin management strategies for almond. The evidence of the low levels of VCG YV36 encountered on almond blossoms and bees during pollination and AF36’s effectiveness in limiting aflatoxin contamination in almond provided additional support for the registration of AF36 with USEPA to use in almond in California.

## Introduction

In California, almonds are grown on over a million and a quarter acres producing over 80% of the annual global almond production [1]. Almond is considered as the top US agricultural export product destined to nut consumption [2]. The crop and its associated industries and by-products (almond milk, almond butter, etc.) are of primary importance to the US economy generating over 21 billion USD of gross revenue [3].

Farmers are able to produce prime quality almonds, in part, through successful control of almond pathogens, particularly fungi [4]. Unfortunately, there are no effective strategies to control certain pathogens, such as aflatoxin-producing fungi, which can occasionally infect and contaminate almond nuts with aflatoxins. Aflatoxins, produced primarily by *Aspergillus flavus* and *A*. *parasiticus*, are mycotoxins that contaminate several economically important crops in California, including almond, fig, and pistachio [5, 6]. There are four major aflatoxins: B_1_, B_2_, G_1_, and G_2_. Both *A*. *flavus* and *A*. *parasiticus* are capable to produce B aflatoxins while from these two species only *A*. *parasiticus* can produce G aflatoxins [4]. Humans and animals are prone to chronic and acute detrimental health effects, including death, due to consumption of aflatoxin-contaminated crops [7–10]. Commodities that exceed aflatoxin thresholds as determined by governmental regulations cannot enter domestic and international markets. Rejection of commodities can cause severe economic losses to growers, packers, and distributors [11, 12].

Aflatoxin contamination in Californian almonds was a minor concern until relatively recently. Increased almond infestation by the navel orangeworm (*Amyelois transitella*) insect pest and severe drought throughout California favored both crop infection by aflatoxin-producing fungi and subsequent aflatoxin formation [5, 13, 14]. The risk of almonds becoming contaminated with aflatoxins is now a constant and serious concern.

Use of several pre- and post-harvest technologies may limit almond aflatoxin contamination; these include agricultural practices, use of insect-resistant varieties, insect control, and sorting, among others [15, 16]. However, these technologies may not be sufficient to result in rendering almonds safe from aflatoxins. A technology not yet exploited in almond production is the use of atoxigenic (non-toxin producing) strains of *A*. *flavus* as biocontrol agents to outcompete (displace) the toxigenic *Aspergillus* fungi. Aflatoxin biocontrol is a safe, commercially proven technology that successfully reduces aflatoxin accumulation before, during, and after harvest in all crops where it is used [6, 17–19]. Two aflatoxin biocontrol products are registered by the United States Environmental Protection Agency (USEPA) in the US and one of them, *Aspergillus flavus* AF36, is used in cotton, maize, and pistachio grown in Arizona, California, New Mexico, and Texas [6, 18]; the other biocontrol product, afla-guard®, is used in maize and groundnut in several US states [20]. In 2015 the USEPA granted California’s fig industry an emergency exemption (Section 18) to utilize AF36 because high aflatoxin levels were expected as a result of drought conditions. The California Fig Advisory Board requested a Section 18 under the clause of the Federal Insecticide, Fungicide, and Rodenticide Act for use in 2016, but it was not approved in a second-time request. However, both the Almond Board of California and the California Fig Advisory Board submitted a petition for the full registration of AF36 (new formulation AF36 Prevail) for use in those two crops. In March 2017 USEPA approved the registration of AF36 Prevail for use in both almond and figs [21] and presently it is pending approval by the California Department of Pesticide Regulations.

The pistachios produced in the US are renowned worldwide for their excellent quality which includes safe aflatoxin levels that are primarily achieved through applications of AF36 [6]. This encouraged the almond industry to pursue AF36’s registration with USEPA to treat the almond crop. Use of AF36 in almond is expected to decrease the aflatoxin contamination of almond in a similar way as in pistachio.

Our laboratory has investigated whether AF36 is appropriate for use in almond. Initial studies revealed that members of the vegetative compatibility group (VCG) to which AF36 belongs, YV36, are also endemic to all almond-growing regions of California (range = 3.5 to 12.6% of recovered *A*. *flavus* isolates) [22]. The natural presence of members of YV36 in almond orchards is particularly important because our laboratory embraces the idea that atoxigenic strains used in biopesticide formulations should be both native to the region where the biocontrol treatment will be applied and superiorly-adapted to the target crop. Furthermore, use of native strains discards environmental concerns posed by the use of exotic fungi [23–25]. Experimental release of AF36 in almond research plots revealed that use of AF36 does not increase either kernel rot nor *Aspergillus* propagule density within and outside of the orchard [22]. A multi-year study revealed that densities of *Aspergillus* spores in AF36-treated and non-treated commercial pistachio orchards remain the same throughout the year, with maxima peaks during the harvest period and low levels during the rest of the year [26]. *Aspergillus* densities in AF36-treated almond orchards throughout the year are expected to be the same as in the absence of treatment. On the other hand, the ability of AF36 to reduce aflatoxin contamination in almond laboratory assays needs to be investigated to determine whether AF36 successfully limits aflatoxin formation on almond nuts when challenged with highly toxigenic *Aspergillus* strains.

Even though using the AF36 biopesticide would improve almond quality, deliberate release of an *A*. *flavus* strain in almond orchards has raised concerns to certain private, public, and academic sectors despite plentiful public information on the safety of this USEPA-approved biological control agent [27, 28]. Honey bee (*Apis mellifera* L.) populations across the US have drastically decreased during the last decade [29]. Honey bees are critically-needed for pollination of many crops, including almond, and growers place bee hives at the border of their orchards, in a ratio of two hives per acre. There is the notion that the use of AF36 may accentuate honey bee population decline. Concerns stem primarily because *A*. *flavus* causes stonebrood and aspergillosis of honey bee larvae and adults, respectively [30]. However, neither *A*. *flavus* nor the genus *Aspergillus* have been implicated as major pathogens of honey bees pollinating almonds in California [31, 32].

Virtually all almond crops in California are pollinated by commercially-managed honey bee colonies which remain in the orchards only during the 3–4 week blossom period (late February–early March) before moving to other locations where their services are needed [31]; most of the beehives are moved to other states while the few beehives remaining in California will be moved to forage in other crops since flowers in and around almond orchards are scarce after the pollination period. *Aspergillus* densities do not become altered after application of AF36 in commercial fields of maize, cottonseed, pistachio, or experimental almond plots [6, 22, 23, 26]. It can thus be expected that, after AF36 applications on almond orchards, honey bees brought in the next season to pollinate the almond crop will not become exposed to *A*. *flavus* propagules in a different manner as in the absence of treatment. Nevertheless, influences of AF36’s release during crop development in contributing to both almond blossom infection and honey bee contamination in the following season have not been formally investigated.

The objectives of this study were to examine *A*. *flavus* communities, particularly of YV36, throughout the almond cropping season in order to (i) determine frequencies of YV36 in almond blossoms, (ii) assess frequencies of YV36 in honey bees collected while pollinating almond blossoms, (iii) determine the optimal period for application of AF36 in almond orchards based on the period in which *Aspergillus* spp. increase in density in almond orchards, and (iv) report AF36 abilities in limiting aflatoxin contamination in laboratory competition experiments with highly toxigenic *Aspergillus* strains. Results from this study provided supportive information for registration of the biopesticide AF36 with the USEPA for use in almond. Now that it is registered, the use of AF36 by almond growers will significantly reduce aflatoxin-contamination of almonds and this will allow to meet domestic and international standards with a low cost, commercially-proven and environmentally-safe technology.

## Materials and methods

### Almond orchard

An almond research plot located at the University of California Kearney Agricultural Research and Extension Center in Parlier, California, was used for these studies. The experimental plot was planted with the cultivars Butte, Carmel, Nonpareil, and Padre in January 2006. The experimental design of the plot is 12 randomized row-blocks with 12 almond trees per row-block. In each row-block, three almond trees of the same cultivar are planted together.

Experimental release of *Aspergillus* strains, both atoxigenic (i.e. AF36) and toxigenic (various *A*. *flavus* and *A*. *parasiticus* strains), occurs on a yearly basis in this plot, including in both 2014 and 2015 (the immediate previous years in which the different samples evaluated in the current study were collected), as part of different experiments that include evaluation of atoxigenic strains to decrease aflatoxin content [22], determining period of almond susceptibility to aflatoxin contamination [33], or assessing influences of both fungicides and insecticides in decreasing aflatoxin content, among others.

### Fungal communities of almond blossoms

Fungal communities of blossoms of the four almond cultivars were examined in both 2015 and 2016 to investigate frequencies of *A*. *flavus*, particularly belonging to the VCG YV36. Four trees per cultivar were randomly selected in four different rows and 30 blossoms per tree were collected and placed into paper bags. In both years, 30 blossoms per tree were collected twice during the third week of February and the second week of March. Immediately after collection, blossoms were placed in sterile plastic screens inside sterile plastic containers used as humid chambers. Sterile water (300 ml) was added to each container to maintain high humidity (approx. 100%). Containers were incubated at 31°C for 7 days. Fungi found growing in the examined blossoms were assigned to their corresponding species based on macroscopic and microscopic characteristics [34, 35]. All *Aspergillus* species isolates were sub-cultured on 5–2 agar [5% V8 juice (Campbell Soup Company, Camden, NJ), 2% Bacto-agar, pH 6.0)[36]] for 7 days (31°C). Isolates were saved as agar plugs of sporulating cultures in 8 ml vials containing 4 ml sterile distilled water until further characterization.

### Fungi contaminating honey bees

Frequencies of *A*. *flavus*, particularly of the VCG YV36, contaminating honey bees were investigated by collecting honey bees attending blossoms of the almond trees mentioned above in both 2015 and 2016. In each year, 120 honey bees were collected randomly by placing one bee per a plastic bag during the first week of March. Honey bees were immediately transferred to the laboratory and sacrificed by storing them at -2°C for 24 h. Honey bees were plated directly on modified rose Bengal agar [37], incubated at 31°C during 3 days in the dark, and the fungal isolates were identified. *Aspergillus* fungi were sub-cultured and saved as described above.

### Fungal communities during almond development

Densities of *A*. *flavus* were examined in green almond fruits of the four cultivars from April to August in 2015. Four trees per cultivar were used on each collection date and the same trees were sampled throughout the study. Fruits were collected on the 1^st^ and 15^th^ day of each month from April through July. In August, fruits were collected on the 1^st^, 8^th^, 15^th^, 22^nd^, and 28^th^ day. Collection dates in August were done on a weekly basis because in previous experiments it was noticed that *Aspergillus* spp. increase in density after the second week of this month. For each tree, during each collection date, 20 random fruits were collected and placed in plastic bags. Then, on each date, five randomly selected fruits were washed with 100 ml sterile water contained in sterile 500 ml Erlenmeyer flasks. After continuous agitation (10 min) in an Eberbach 6010 reciprocating shaker (Ann Arbor, MI), 100 μl of the wash were combined with 9.9 ml of sterile water. Dilutions were vortexed and 100 μl aliquots were plated on acidified potato dextrose agar (APDA) [38], six plates per dilution. Almost all (> 98%) of the contaminating fungi were assigned to their corresponding genus.

### Fungal communities during pistachio development

Because pistachio and almond plots are frequently planted in close proximity, fungal communities associated with pistachio throughout the growing season were examined in commercial orchards in 2015. Pistachio fruits were periodically collected from April 18^th^ to September 8^th^. On each date, three samples of 10 fruit clusters were randomly collected. Ten randomly selected fruits were washed as described above. Dilution plating and incubation were conducted as described above. Most (>95%) of the contaminating fungi were assigned to their corresponding species based on macroscopic and microscopic characteristics.

### Vegetative compatibility analyses

All *A*. *flavus* isolates recovered from both almond blossoms and honey bees were tested for membership in YV36, the VCG to which the biopesticide AF36 belongs. Nitrate non-utilizing (*nit*) mutants were obtained by plating spore suspensions (approximately 1,000 spores in 15 μl) of isolates on SEL agar (Czapek-Dox broth, 25 g KClO_3_, 50 mg rose Bengal, and 2% Bacto-agar (Difco Laboratories Inc., Detroit, MI), per liter, pH 7.0) into 3 mm central wells cut into agar. Plates were incubated for up to one month (31°C). Spontaneous auxotrophic sectors were transferred onto MIT agar (Czapek-Dox broth, 15 g KClO_3_, and 2% Bacto-agar per liter, pH 6.5), and incubated for 3 days (31°C) to stabilize the mutants. Mutants were then grown on 5–2 agar for 7 days (31°C). VCG membership was determined by using previously described [39] testers of VCG YV36, ATCC 96045 and ATCC 96047, and following previously described protocols [6]. Briefly, fungal suspensions (15 μl) of the two testers and the *nit* mutant from the isolate under evaluation were seeded independently into one of three 3 mm wells spaced 1 cm apart in a triangular pattern on starch agar (36 g dextrose, 20 g soluble starch, 3 g NaNO_3_, 2% Bacto-agar per liter, pH 6.0 [40]) and incubated for 7 days (31°C, dark). Formation of prototrophic growth in the zone of hyphal interaction between a *nit* mutant and either of YV36 testers indicated membership in VCG YV36.

### Co-inoculation of AF36’s active ingredient with toxigenic *Aspergillus* isolates on viable almond kernels

The ability of AF36’s active ingredient in limiting aflatoxin accumulation when co-inoculated independently with highly toxigenic isolates of either *A*. *flavus* or *A*. *parasiticus*, isolates 2A1L-11 and 4C1P-11, respectively, was investigated in competition laboratory assays. Both toxigenic isolates produce large quantities of aflatoxin in chemically defined media, as well as viable almond kernels (Picot and Michailides, *unpublished results*). For inoculum preparation, isolates were grown on 5–2 agar for 7 days at 31°C. Conidia were collected with a sterile cotton swab and suspended in sterile deionized water. Conidial suspensions were quantified using a hemocytometer and diluted to a final inoculum concentration of 1.75 × 10^6^ conidia/ml. Sterile glass vials (20 ml) containing approximately 5 g (about 6 to 7 kernels) of mature, living almond kernels previously surface-disinfested by submersion in hot water (80°C, 45 s) were either co-inoculated with a combination of a toxigenic and AF36 isolate or inoculated with a toxigenic isolate alone. Each vial was inoculated with a fungal suspension containing approximately 350,000 conidia per g of almonds, which was previously combined with the appropriate amount of distilled water to bring almond moisture content to 25%. The initial almond moisture content was 6%. The adjusted suspensions were vortexed and distributed evenly on the surface of almond kernels inside the sterile glass vials. When co-inoculation occurred, equal conidia amounts from the two isolates were used. Almond-containing vials inoculated with sterile water served as non-inoculated controls. After inoculation, flasks were covered with sterile plastic caps, positioned in a crisper into a randomized design, and incubated at 31°C for 7 days. After the incubation period, the experiment was terminated by adding 30 ml of 60% methanol. Aflatoxins were extracted following the official analysis method of the Association of Official Analytical Chemists [41]. Aflatoxins were quantified using an HPLC Hewlett Packard 1050 as previously described in other studies by our laboratory [6]. Four replicates per treatment were used. Each replicate consisted of a single glass vial containing almonds. The experiment was repeated twice.

### Statistical analysis

Data were summarized and analyzed using Statistix 10 (Analytical Software, Tallahassee, FL). The effect of the cultivar on the percentage of isolation of each fungal species or the differences on isolations percentage among fungal species were evaluated using Zar’s test of multiple comparisons of proportions [42]. Non-parametric Friedman’s test was used to study the population of fungal species from almond fruits. After that, differences among almond cultivars were determined using Dunn’s test with a Bonferroni adjustment at *P* = 0.05.

Species diversity for each almond cultivar on each collection date was calculated based on Shannon-Weiner diversity index (*H*) with *Pi* values: $H=-\sum_{i=1}^{S}Pi*lnPi$ where *Pi* is the proportion for the *i*th species and *S* is the total number of species in the population (species richness). Equitability (*J*) was calculated as: $\frac{H}{lnS}$. *J* assumes values between 0 and 1. The closer *J* gets to 1, the more equitable is the population [43].

The Welch's *t*-test, or unequal variances *t*-test, was used to compare aflatoxin concentrations between almond kernels inoculated with the toxigenic isolates alone and almond kernels co-inoculated with both toxigenic and atoxigenic isolates.

Aflatoxin reductions by AF36 were calculated as [1 –aflatoxin content in almonds co-inoculated with a toxigenic isolate and AF36 isolate/ aflatoxin content in almonds inoculated by the toxigenic isolate alone] x 100 as described by Probst and co-workers [24].

## Results

### Blossoms

In each year, results of the two collection dates were relatively similar (*Df* = 1; Chi-Square = 0.1230; *P* = 0.7257) and were combined for analyses. In total, 900 blossoms were examined during both years. Overall, *Alternaria* spp. was the most commonly identified group (77.8%), followed by *A*. *flavus* (2.1%), *Fusarium* spp. (2.0%), *A*. *niger* (1.5%), and *Penicillium* spp. (0.2%); there was no fungal growth in 16.4% of blossoms. The population of *Alternaria* species was significantly lower (*Df* = 3; Overall Chi-Square = 33.3; *P* = 0.0001) in blossoms of the cultivars Padre and Nonpareil than in the blossoms of the other cultivars. Furthermore, the cultivar Butte had higher (*Df* = 3; Overall Chi-Square = 15.75; *P* = 0.0013) frequencies of *A*. *niger*. Frequencies of *A*. *flavus*, *Fusarium* spp., and *Penicillium* spp. were similar (*Df* = 3; Chi-Square < 7.8147; *P values* > 0.05) among all the cultivars. Noteworthy, in 2015 none of the examined blossoms harbored *A*. *flavus*, or any other aflatoxin-producing species. In 2016, 4.0% of blossoms were contaminated with *A*. *flavus*. Vegetative compatibility analyses revealed that only one out of 18 *A*. *flavus* isolates associated with the blossoms belong to VCG YV36.

### Honey bees

Results of the two honey bee collections, one per year, were relatively similar and combined for analyses. *Alternaria* species was the most commonly identified group (21.4%), followed by *Rhizopus* spp. (20.8%), *A*. *niger* (20.0%), *Fusarium* spp. (19.4%), *A*. *flavus* (8.5%), *Penicillium* spp. (5.3%); *A*. *fumigatus* (2.4%), *Trichoderma* spp. (1.2%), and *Botrytis* spp. (1.2%). Overall, for all dominant genera (i.e., *Alternaria*, *Rhizopus*, *Aspergillus*, and *Penicillium*) there were no significant differences (*Df* = 2; Chi-square < 5.9915, *P values* > 0.05) in frequencies between the two examined periods. VCG testing revealed that none of the recovered *A*. *flavus* isolates belong to VCG YV36.

### Developing almonds

**Fig 1:**
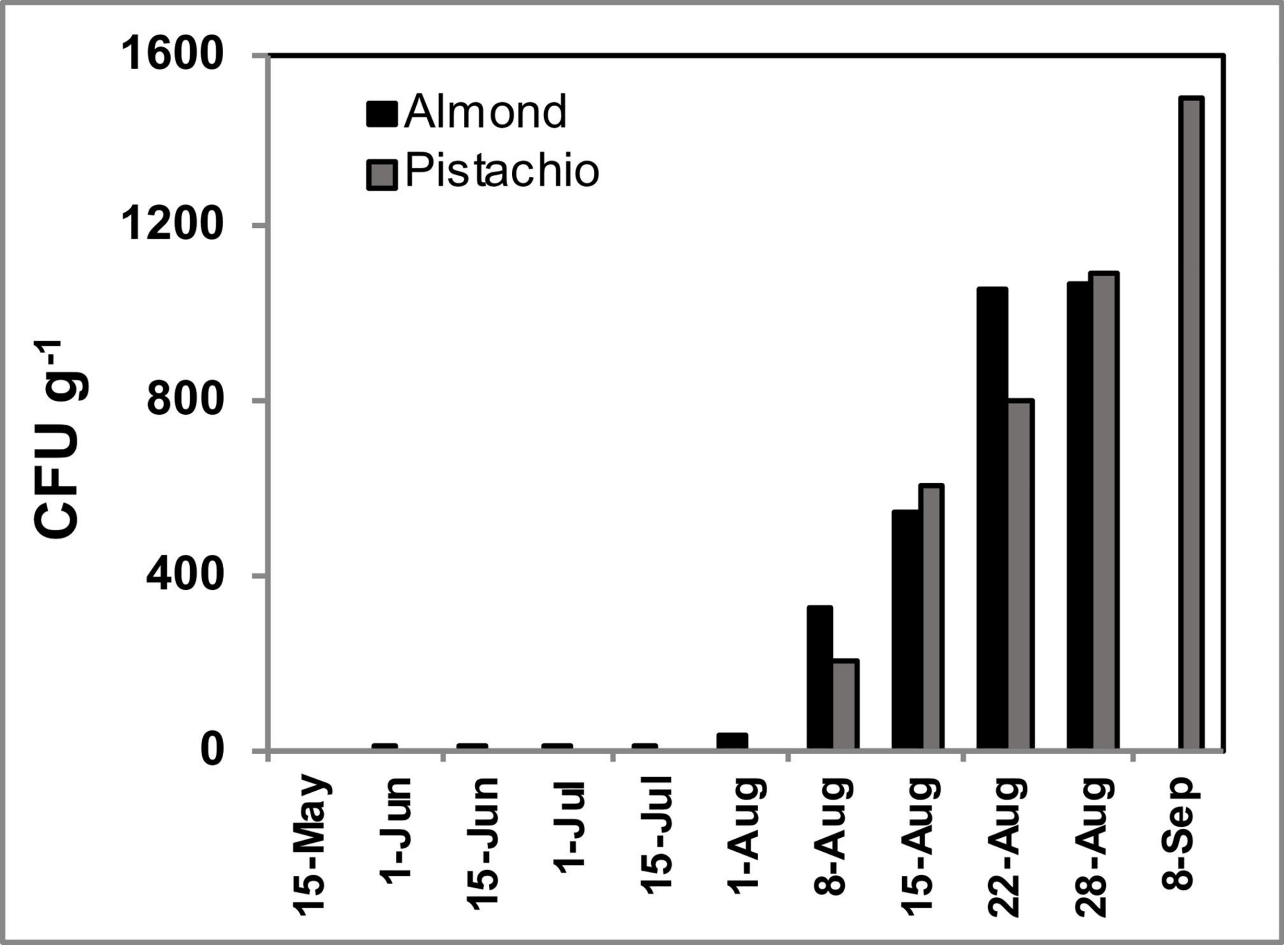
Fungal densities of *Aspergillus* spp. in almond and pistachio orchards at the Kearney Agricultural Research and Extension Center in Parlier, CA. In both crops, *Aspergillus* spp. fungi increase after the second week of August. Developing almond fruits were not examined in September 8^th^.

During the course of the study over 45,000 fungal isolates belonging to 17 genera were enumerated. The most commonly identified genera were *Acremonium* (52.9%), *Cladosporium* (5.4%), *Aureobasidium* (4.1%), *Aspergillus* (3.1%), *Fusarium* (1.0%), *Alternaria* (0.3%), *Penicillium* (0.3%), and different species of yeasts (32.5%). The Botryosphaeriaceae species and species belonging to the genera *Botrytis*, *Coniothyrium*, *Epicoccum*, *Colletotrichum*, *Ophiostoma*, *Phomopsis*, *Rhizopus*, and *Trichoderma* were detected at frequencies of ≤ 0.2%. Only 1.1% of the recovered fungi were not assigned to a corresponding species, mainly because they did not produce any spores in culture media. Relatively low densities of the genus *Aspergillus* (0 to 6 CFU/g), the main subject of the current study, were detected until August 8^th^ (Fig 1). Densities of *Aspergillus* spp. increased linearly over time during August (Y = 284.5X – 237.9; *R*^*2*^ = 0.9516; *P* = 0.0008). Significantly (Friedman’s test; *F* = 17.24; *Df* = 3.3; *P* = 0.0001) more *Aspergillus* spp. fungi were recovered from Nonpareil cultivar while Butte and Padre cultivars harbored significantly (Dunn’s test; *P* > 0.05) less *Aspergillus* propagules. Carmel formed an intermediate group (Dunn’s test; *P <* 0.05) between the other two according to the percentage of *Aspergillus* isolation. This test also showed that the *Aspergillus* inoculum increased across the season with maxima peaks during August.

**Fig 2:**
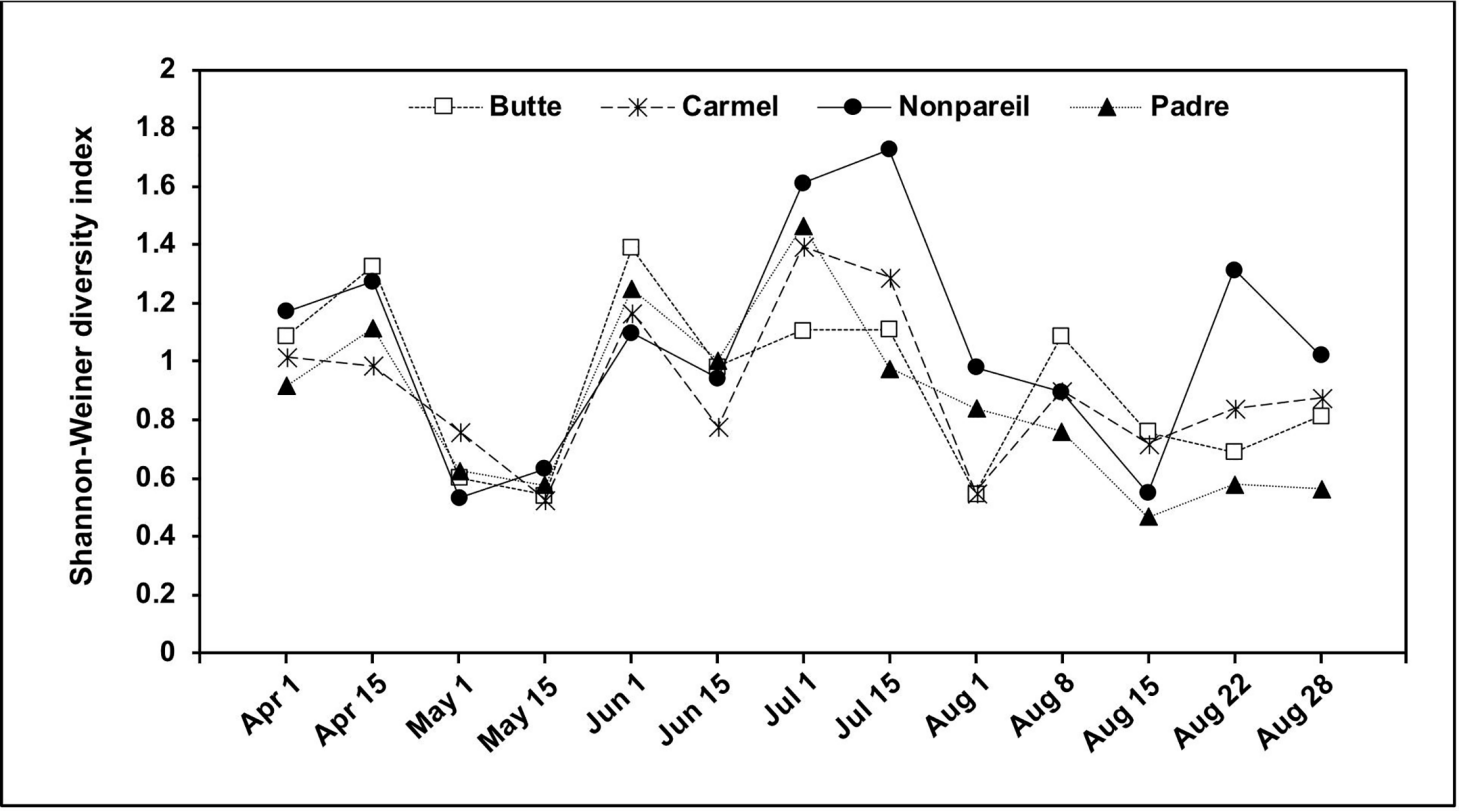
Shannon-Weiner diversity indices of fungal species detected in four almond cultivars during the course of the study in an experimental orchard at the Kearney Agricultural Research and Extension Center in Parlier, CA.

The overall Shannon-Weiner diversity indices were 0.857, 0.908, 0.925, and 1.050 for Padre, Carmel, Butte, and Nonpareil cultivars, respectively. Diversity indices fluctuated among the examined cultivars across the growing season but none of the cultivars exhibited consistently lower or higher diversity on any given date (Fig 2). Overall, fungal diversity was highest from July 1^st^ to 15^th^ for all studied cultivars except for Butte, in which the highest value was detected on June 1^st^.

### Developing pistachios

Over 50,000 isolates were enumerated during the course of the study. The genera *Aureobasidium* (25.4%), *Cladosporium* (24.6%), *Alternaria* (19.2%), *Penicillium* (7.6%), *Aspergillus* (2.3%), *Botrytis* (1.7%) and black and white yeasts (15.2%) were recovered at relatively high frequencies in the examined pistachio fruits during certain periods or throughout the whole season. Other less frequently recovered genera included *Epicoccum*, *Fusarium*, *Mucor*, *Rhizopus*, and *Paecilomyces* with less than 1% of the population. Throughout the course of the study, densities of *Alternaria* and yeasts changed moderately while densities of *Botrytis*, *Penicillium*, and *Aspergillus* changed considerably. Propagules of *Aspergillus* spp. increased to significant proportions during the first week of August (average 200 CFU/g) and continued to increase throughout that month (average 1,500 CFU/g; Fig 1).

### Ability of AF36 to reduce aflatoxin contamination when challenged with toxigenic strains on almond substrate

**Fig 3:**
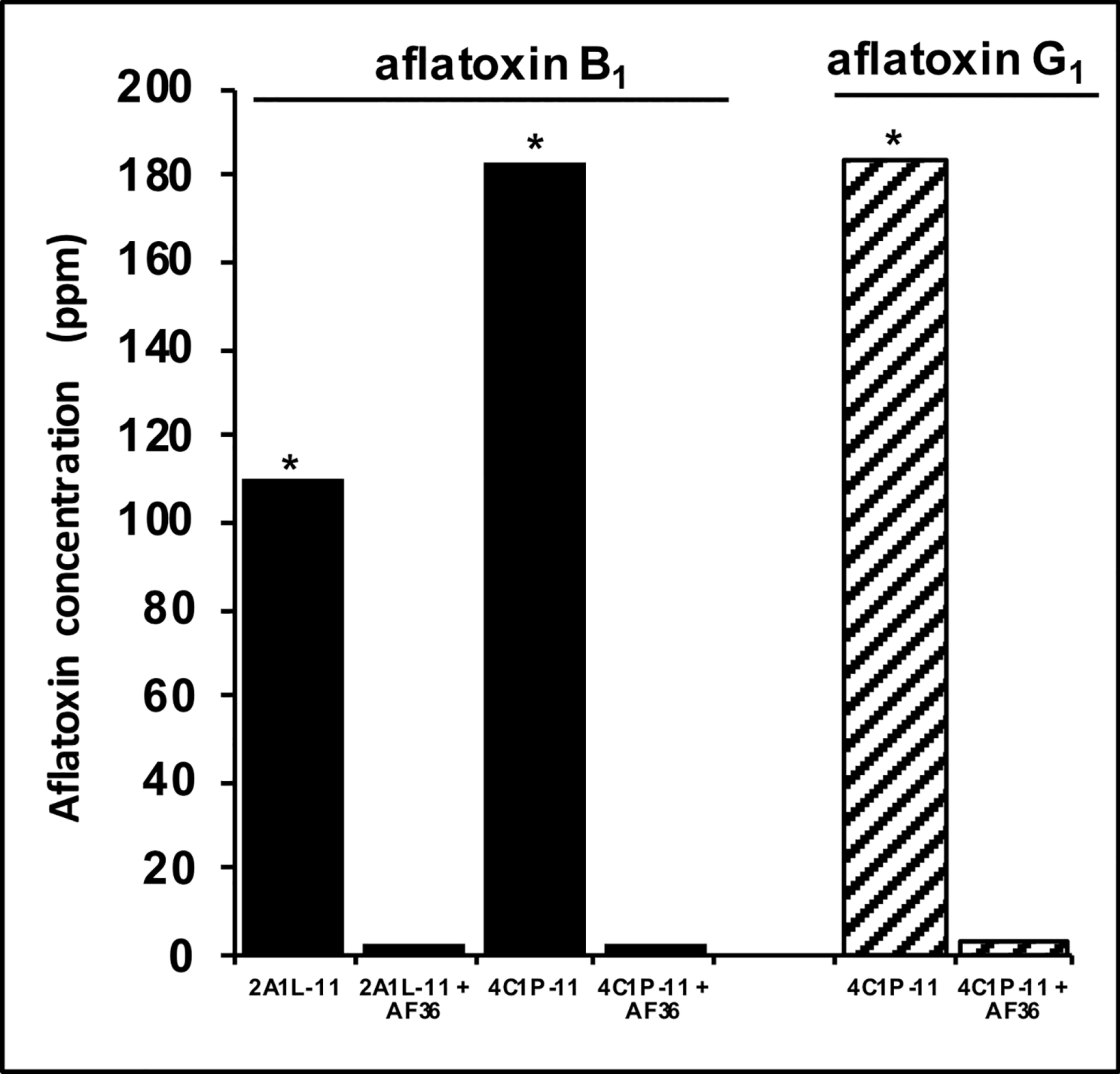
Ability of *Aspergillus flavus* AF36’s active ingredient to reduce aflatoxin accumulation in viable almonds when co-inoculated with toxigenic *A*. *flavus* 2A1L-11 or *A*. *parasiticus* 4C1P-11 isolates. Both toxigenic isolates are native to California almond agroecosystem and produce large aflatoxin quantities in several substrates. Asterisks indicate significant differences (Welch's *t*-test; α = 0.05) in aflatoxin concentrations between toxigenic isolates inoculated alone and that isolate co-inoculated with AF36. Almond fermentations co-inoculated with AF36 accumulated over 95% less aflatoxins in comparison to almonds inoculated with a toxigenic isolate alone.

Almonds co-inoculated with AF36 and highly toxigenic strains of either *A*. *flavus* or *A*. *parasiticus* had >95% less aflatoxins than almonds inoculated with toxigenic strains alone (Fig 3). AF36 was highly efficient in reducing both aflatoxin B_1_ (by both *A*. *flavus* and *A*. *parasiticus*) and G_1_ (by *A*. *parasiticus*).

## Discussion

This study describes frequencies of the fungus *A*. *flavus* throughout the almond growing season. In addition, we investigated frequencies of members of vegetative compatibility group (VCG) YV36 in almond blossoms and honey bees pollinating the blossoms of this crop. YV36 is the VCG to which the aflatoxin biocontrol agent *Aspergillus flavus* AF36 belongs [44]. This is the first study in which frequencies of an *A*. *flavus* genotype are monitored throughout this critical period for almond production. Finally, the ability of AF36’s active ingredient to limit aflatoxin contamination in almond substrate was investigated. Our results indicate that frequencies of YV36, and *A*. *flavus* in general, are low and infrequent during almond blossoming-pollination period and that densities of *Aspergillus* spp. remain relatively low in developing almond fruits until the latter stages of nut maturation, in August. In addition, AF36 was found to effectively limit aflatoxin contamination when challenged with highly toxigenic *Aspergillus* strains during laboratory competition assays in almond kernels (Fig 3).

The species *A*. *flavus* is a pathogen of plants, animals, and insects [45]. Its role as a plant pathogen has received considerable attention because it contaminates a wide range of crops with aflatoxins, which pose serious health threats to both humans and animals even at very low concentrations [10]. Aflatoxin contamination of susceptible crops is common in regions with tropical and subtropical climates [46]. In addition, susceptible crops that are irrigated can be subjected to hot and humid environments, favoring aflatoxin contamination. Furthermore, during the last decade, susceptible crops have suffered from aflatoxin contamination events in areas traditionally free of contamination because of drought conditions driven by climate change which predispose crops to infection by aflatoxigenic fungi [47, 48].

Almond, a crop of great economic importance for both California and the US, poses increased risk of aflatoxin contamination due to recent drought conditions [5, 49]. In the past, the only effective preventive measure to limit aflatoxin contamination in almond was to control the navel orangeworm, which creates wounds for infection when larvae bore into the nutmeat and carries propagules of aflatoxin-producing species. For other commercial crops a very effective approach to reduce aflatoxin contamination is the use of atoxigenic *A*. *flavus* strains as biocontrol agents to competitively displace aflatoxin-producers during crop development [6, 18].

The biocontrol agent *Aspergillus flavus* AF36 is one of the two atoxigenic biopesticides registered with the USEPA that until early 2017, was approved for use in cotton, maize, and pistachio. These crops can be treated yearly at label rates per hectare with AF36 in Arizona, California, New Mexico, and Texas [27, 28]. Fortunately, this biopesticide was approved by USEPA in March 2017 for use in almond and fig crops [21]. The active ingredient fungus of AF36 belongs to VCG YV36. This VCG is highly associated with almond throughout California [22]. The high number of rejected almond loads exported to Europe due to exceeding aflatoxin tolerance levels (particularly in 2007), a high association of YV36 with almond, and successful aflatoxin reductions by AF36 in other crops sparked an interest in the almond industry to use AF36 to reduce aflatoxin contamination of almonds. In order to obtain USEPA approval to use AF36 in almond, several requirements needed to be addressed and satisfied, including providing evidence that the use of AF36 in almond poses no threat to the critically endangered honey bee populations. Certain academic and governmental sectors feared that field release of an organism with potential to negatively affect honey bees will accentuate honey bee decline [29, 30] in California, where several crops, including almond, rely on honey bees for pollination On the other hand, *A*. *flavus* is not a major pathogen of honey bees pollinating almonds in California [31, 32]. The application of *Aspergillus flavus* AF36 in the field is planned for late June or during July, long after the pollination of almond and the flights of honey bees in almond orchards have been completed. By June–July, most commercially-managed honey bees (which virtually pollinate all almond crops in California) have been transported to other states to provide their pollination services to other crops [31].

Only 2.1% of examined blossoms of the four almond cultivars were contaminated with *A*. *flavus*. This occurred regardless of continuous experimental release of both atoxigenic and toxigenic *Aspergillus* fungi during previous cropping seasons [22, 33] and relatively high natural *Aspergillus* densities throughout California’s Central Valley [5, 6, 16]. Low (< 3%) frequencies of *Aspergillus* spp. in almond blossoms were previously detected in several unrelated studies in which thousands of blossoms were screened for fungal contaminants (Michailides, *unpublished results*). Results from previous and the current study suggest that *Aspergillus* spp. remain predominantly inactive during almond blossoming due to the low temperatures prevalent during that period (late February and during March in the northern hemisphere). Furthermore, *A*. *flavus* was detected only in blossoms collected in 2016 with only one isolate belonging to VCG YV36. Also, we did not observe sporulating *Aspergillus* spp. on blossoms under the field conditions.

Relatively low frequencies of *A*. *flavus* were detected in honey bees collected while pollinating almond blossoms (8.5% of the examined fungi) with none of those isolates belonging to YV36. Other airborne species (e.g. *Alternaria* spp., *Rhizopus* spp., and *A*. *niger*) occurred at higher frequencies in almond blossoms. It has been reported that other organisms contribute to honey bee disorders to a greater extent than *A*. *flavus* [29, 32, 50–53] even though *Aspergillus* species have been reported as pathogens of both larvae and adult honey bees [30]. Low frequencies of *A*. *flavus* in both blossoms and honey bees indicate both that honey bees do not become exposed to *A*. *flavus*, or are exposed at relatively low densities, while pollinating almond blossoms. Since frequencies of *A*. *flavus* in blossoms were found to be low, the contamination of honey bees with *A*. *flavus* is highly likely to originate from sources unrelated to the almond pollination process.

Thousands of fungal isolates were recovered during the examination of communities associated with developing almond fruits. Several genera were recovered throughout five consecutive months. In general, higher fungal diversity was found from June to late July (Fig 2), when nuts start maturing in California; during that period several fungal genera dominate the communities, but *Aspergillus* species occur at either low densities or do not occur at all (Fig 1). Our results indicate that *Aspergillus* species contaminate almond kernels more frequently during the final development stages, i.e. from hull split until almonds are mature, the most susceptible period for fungal infection and aflatoxin formation [33], which occurs throughout August. That is the period in which navel orangeworm is associated with almond crops and creates larval boring wounds that facilitate infection by aflatoxin-producing fungi that the insect typically carries; when navel orangeworm is not properly controlled, high aflatoxin contamination occurs [16]. When the current study was designed, we expected to find *Aspergillus* spp. throughout most of the almond developmental stages. Interestingly, *Aspergillus* spp. remained predominantly inactive from blossoming until hull split. In a similar manner, densities of *Aspergillus* spp. in developing pistachio fruits increased until late August close to the harvest period. Developing pistachio nuts also interact with *Aspergillus* spp. only during a small portion of fruit development/maturation processes. It is currently unknown which biotic and/or abiotic factors trigger reproduction rates in *Aspergillus* spp. inhabiting almond and pistachio orchard soils during the later stage of crop development.

For pistachio, it has been determined that the optimal period for application of AF36 is by the middle of July [6]. Based on our findings in this and previous studies (Doster and Michailides, *unpublished results*) that *Aspergillus* spp. increase in density in the almond orchard during early August the optimal application date for AF36 should be 2–3 weeks before the hull splitting period. Atoxigenic application during the proposed pre-hull-split (middle of July) period should be sufficient for the atoxigenic strain to reproduce on the delivery substrate (i.e. sterile wheat or sorghum grain), colonize organic matter sources present in the orchard, and then move to the maturing almond fruit to colonize the substrates that otherwise fungi residing in orchard soils and/or neighboring areas would do during the most susceptible period to aflatoxin contamination, second week of August and until harvest [33].

*Aspergillus* densities in soils and crops of maize, cotton, and pistachio treated with AF36 are not significantly different from those occurring in non-treated fields [6, 18, 26]. Experimental use of AF36 on almond research plots of the University of California also resulted in unaltered *Aspergillus* densities on both soil and kernels when comparing treated to non-treated plots [22]. Our results indicate that application of AF36 and other *Aspergillus* genotypes in the research orchard during late July does not result in increased densities of *A*. *flavus* during the pollination period the next calendar year, in February and March. In the research almond orchard, densities of *Aspergillus* spp. decrease significantly after the cropping season (end of September) regardless of the deliberate dispersal of *Aspergillus* genotypes. Therefore, application of AF36 during the summer should not negatively affect honey bees pollinating almond blossoms during the next cropping season, in late winter.

Use of AF36 in Californian almond orchards is feasible because of both its adaptation to almond and its ability to substantially reduce aflatoxin accumulation in almond nuts [6, 22]. In addition, the current study reports findings that were submitted to USEPA that supported registration AF36 for use in almond orchards. Low densities of both *A*. *flavus* and VCG YV36 contaminating almond blossoms and honey bees indicate that use of AF36 pose a low risk to honey bees transported to California to pollinate almond during a short period [31]. Results from both previous studies [6, 18, 22] and the current one facilitated rapid USEPA registration of this biological agent for use in almond for prevention of aflatoxin contamination before, during, and after harvest. Use of AF36 by almond growers will allow production of almonds with safe aflatoxin levels. Almond from treated fields will meet domestic and international standards by using a low cost, commercially-proven, and environmentally-safe technology.
